# The Nuclear DNA Sensor IFI16 Indiscriminately Binds to and Diminishes Accessibility of the HSV-1 Genome to Suppress Infection

**DOI:** 10.1128/msystems.00198-22

**Published:** 2022-05-16

**Authors:** Timothy R. Howard, Krystal K. Lum, Michelle A. Kennedy, Ileana M. Cristea

**Affiliations:** a Department of Molecular Biology, Princeton University, Princeton, New Jersey, USA; NIAID, NIH

**Keywords:** ATAC-seq, ChIP-seq, DNA sensor, HSV-1, IFI16, PRM, proteomics, targeted mass spectrometry, virus infection, virus-host interactions

## Abstract

Human cells identify invading pathogens and activate immune signaling pathways through a wide array of pattern recognition receptors, including DNA sensors. The interferon-inducible protein 16 (IFI16) is a nuclear DNA sensor that recognizes double-stranded DNA from a number of viral sources, including genomes of nuclear-replicating viruses. Among these is the prevalent human pathogen herpes simplex virus 1 (HSV-1). Upon binding to the HSV-1 DNA genome, IFI16 both induces antiviral cytokine expression and suppresses virus gene expression. Here, we used a multiomics approach of DNA sequencing techniques paired with targeted mass spectrometry to obtain an extensive view of the interaction between IFI16 and the HSV-1 genome and how this binding affects the viral DNA structure and protein expression. Through chromatin immunoaffinity purification coupled with next-generation DNA sequencing (ChIP-seq), we found that IFI16 binds to the HSV-1 genome in a sequence-independent manner while simultaneously exhibiting broad enrichment at two loci: UL30, the viral DNA polymerase gene, and US1 to US7. The assay for transposase-accessible chromatin with sequencing (ATAC-seq) revealed that these two regions are among the most accessible stretches of DNA on the genome, thereby facilitating IFI16 binding. Accessibility of the entire HSV-1 genome is elevated upon IFI16 knockout, indicating that expression of IFI16 globally induces chromatinization of viral DNA. Deletion of IFI16 also results in a global increase in the expression of HSV-1 proteins, as measured by parallel reaction monitoring-mass spectrometry of viral proteins representing 80% of the HSV-1 genome. Altogether, we demonstrate that IFI16 interacts with the HSV-1 genome in a sequence-independent manner, coordinating epigenetic silencing of the viral genome and decreasing protein expression and virus replication.

**IMPORTANCE** Mammalian host defense against viral infection includes broad-acting cellular restriction factors, as well as effectors of intrinsic and innate immunity. IFI16 is a critical nuclear host defense factor and intrinsic immune protein involved in binding viral DNA genomes, thereby repressing the replication of nucleus-replicating viruses, including the human herpes simplex virus 1. What has remained unclear is where on the viral genome IFI16 binds and how binding affects both viral DNA structural accessibility and viral protein expression. Our study provides a global view of where and how a nuclear restriction factor of DNA viruses associates with viral genomes to exert antiviral functions during early stages of an acute virus infection. Our study can additionally serve as a systems-level model to evaluate nuclear DNA sensor interactions with viral genomes, as well as the antiviral outcomes of transcriptionally silencing pathogen-derived DNA.

## INTRODUCTION

The ability of cells to identify and respond to invading pathogens is crucial for the maintenance of cellular and tissue homeostasis. Viruses with double-stranded DNA (dsDNA) genomes that replicate within the nucleus, like herpesviruses, constitute a significant threat to human health. To counter these infections, host cells utilize nuclear DNA sensors that bind virus dsDNA (vDNA) within the nucleus and initiate intrinsic and innate immune signaling pathways. To date, four proteins have emerged as nuclear DNA sensors with critical roles in identifying and reacting to virus infection: interferon-inducible protein 16 (IFI16) (1–3), interferon-inducible protein X (IFIX, also known as PYHIN1) (4–6), cyclic GMP-AMP synthase (cGAS) (7–10), and heterogenous nuclear ribonucleoprotein A2/B1 (hnRNPA2B1) (11). As pathogen-specific moieties have yet to be identified on viral DNA in the nucleus, the prevailing paradigm is that nuclear DNA sensors must discern between structurally similar host and pathogenic DNA. Characterizing the interaction between nuclear DNA sensors and virus DNA is essential for building an understanding of how these proteins drive immune signaling platforms in response to virus infections. Additionally, as vaccine technologies shift toward utilizing nucleic acids to facilitate the production of antigens (12–14), it is crucial that we are able to predict how nuclear DNA sensors react to these incoming foreign molecules.

As the first discovered nuclear DNA sensor, investigations into the role of IFI16 during virus infections continue to lay the foundation for identifying and characterizing nuclear DNA sensors. Belonging to the PYHIN family of proteins, IFI16 contains an N-terminal pyrin domain (PYD) that mediates homotypic protein interactions (15) and two C-terminal HIN-200 (hematopoietic expression, interferon-inducible nature, and nuclear localization) domains, HINa and HINb, which bind to dsDNA (16, 17). During infection with herpes simplex virus 1 (HSV-1), the virus capsid docks at the nuclear pore complex and injects the naked dsDNA genome into the nucleus (18). Following this event, IFI16 relocalizes from the nucleolus to the nuclear periphery, where it binds to the vDNA via the HIN domains and oligomerizes in a PYD-dependent manner (1, 9, 19, 20). The binding of IFI16 to vDNA results in the induction of antiviral cytokines, at least in part through the STING-TBK1-IRF3 signaling axis (1, 2, 19–21).

Research in recent years has focused on how DNA sensors target pathogenic DNA and avoid binding to host DNA, which could spuriously activate immune signaling. Structural studies determined that both IFI16 HIN domains possess consecutive oligonucleotide/oligosaccharide binding (OB) folds (16, 22, 23). Further crystallographic investigations revealed that these OB folds bind to DNA via weak electrostatic interactions, allowing sequence-independent recognition of dsDNA (17, 24). *In vitro*, the binding of HINa to virus dsDNA was weaker than that of HINb and HINab (1). Further investigations showed that HINa contacts the DNA backbone of one strand of dsDNA at a time, allowing it to also bind single-stranded DNA (ssDNA) (24). In contrast, HINb contacts both strands of dsDNA simultaneously (17). Additionally, the two IFI16 HIN domains play distinct roles during DNA sensing, with HINa dampening and HINb promoting IFN-β expression (24). These findings suggest independent roles for the two IFI16 HIN domains, which increases the repertoire of possible IFI16 targets and allows finer tuning of downstream immune signaling.

Cooperative assembly of IFI16 molecules, driven by PYD oligomerization, also significantly contributes to effective binding of DNA (25) and the antiviral capacity of IFI16 (19, 20). Optimal IFI16 oligomerization efficiency is achieved along 150 bp of free dsDNA, with one IFI16 molecule occupying 15 bp of DNA (25). Moreover, IFI16 molecules one-dimensionally track along dsDNA before oligomerizing, and host nucleosomes act as barriers that prevent cooperative assembly of IFI16 supramolecular structures (26). These studies have led to the view that IFI16 avoids accidental immune activation by failing to oligomerize upon the tightly packaged host dsDNA.

Beyond its ability to induce cytokine expression, IFI16 exerts another antiviral function upon its binding to vDNA, i.e., by restricting viral gene expression (20, 27–29). It is hypothesized that, to suppress gene expression and virus replication, IFI16 modulates the chromatin state of the HSV-1 genome. Chromatin immunoaffinity purification (ChIP)-quantitative PCR (qPCR) experiments showed that IFI16 knockout results in decreased association of the heterochromatin marker H3K9me3 with three tested HSV-1 genomic loci (promoters of ICP4, ICP27, and ICP8), in conjunction with an increase in the euchromatin marker H3K4me3 (28). A subsequent study recapitulated these results and further revealed that IFI16 expression reduces TATA binding protein, Oct1, and RNA polymerase II occupancy at HSV-1 transcriptional start sites (promoters of ICP0, ICP4, ICP8, gB, and US11) (29). That study also utilized IFI16 ChIP-qPCR to investigate the binding of IFI16 to the HSV-1 genome at these five loci. Similar degrees of DNA amplification following IFI16 isolation suggested that IFI16 binds to vDNA without regard for DNA sequence and targets all temporal classes of HSV-1 genes. However, the authors observed minor cell type-dependent differences in IFI16 binding: in human foreskin fibroblasts (HFF), IFI16 associated most strongly with the US11 promoter, while this association was the weakest in U2OS cells (29).

Altogether, the findings of previous studies suggest that IFI16 binds vDNA in a sequence-independent manner and suppresses viral gene expression via chromatin remodeling. However, the ChIP-qPCR experiments that support these claims face two major limitations. First, reliance on qPCR limits the measured association between IFI16 and vDNA to a few hundred base pairs, which is only a fraction of the 152-kbp HSV-1 genome. Second, instead of a direct readout, the association of vDNA with H3 chromatin markers acts as a proxy for the vDNA chromatin state. As such, how IFI16 interacts with the HSV-1 genome and drives changes in the chromatin landscape to suppress viral gene expression remains largely unexplored, and several unanswered questions persist. Does IFI16 bind evenly along the vDNA without regard for sequence motifs? Could protein-protein interactions with virus or host factors drive IFI16 enrichment at specific loci, such as viral gene promoters? Does IFI16 binding globally promote heterochromatinization of the virus genome, or do local effects occur where IFI16 binding is enriched? Further, would such local changes in vDNA chromatinization manifest downstream as IFI16-mediated suppression of specific genes?

Here, we used a systems-level approach to address these questions, and we present the most detailed characterization to date of a nuclear DNA sensor binding to vDNA and suppressing viral gene expression. Specifically, we used ChIP coupled with next-generation DNA sequencing (ChIP-seq) to demonstrate that IFI16 binds along the entire HSV-1 genome in a sequence-independent manner, additionally finding that IFI16 is enriched at two loci critical for vDNA replication: the genes UL30 and US1 to US7. We complemented these findings using the assay for transposase-accessible chromatin with sequencing (ATAC-seq) to investigate the vDNA chromatin landscape during early HSV-1 infection. We established that UL30 and the unique short (U_S_) region are the most transposase-accessible regions of the HSV-1 genome. The enrichment of IFI16 at these loci suggests that increased binding to vDNA is driven by local genome accessibility, resulting in cooperative assembly of IFI16 molecules. It is possible that, given the proximity of these regions to the origins of replication, the increased genome accessibility and subsequent IFI16 enrichment are consequences of viral processes such as vDNA replication. Further, knockout of IFI16 enhances transposase accessibility across the entire HSV-1 genome, indicating that expression of IFI16 globally promotes chromatinization of the vDNA, regardless of the relative enrichment of IFI16 at specific loci. We then leveraged the targeted proteomics technique known as parallel reaction monitoring-mass spectrometry to show that IFI16-mediated chromatinization of vDNA is accompanied by global suppression of virus protein expression. Altogether, we demonstrate that IFI16 binds to the HSV-1 genome without preference for DNA sequence, and this interaction instigates concomitant global changes in the vDNA chromatin landscape and reductions in virus protein levels. Our multiomics study presents the most complete view of a nuclear DNA sensor binding to viral dsDNA, providing insights into how IFI16 represses viral replication by altering the accessibility of the virus genome to host and viral factors.

## RESULTS

### IFI16 binds to the HSV-1 genome irrespective of DNA sequence and is enriched at the genes UL30 and US1–US7.

**FIG 1:**
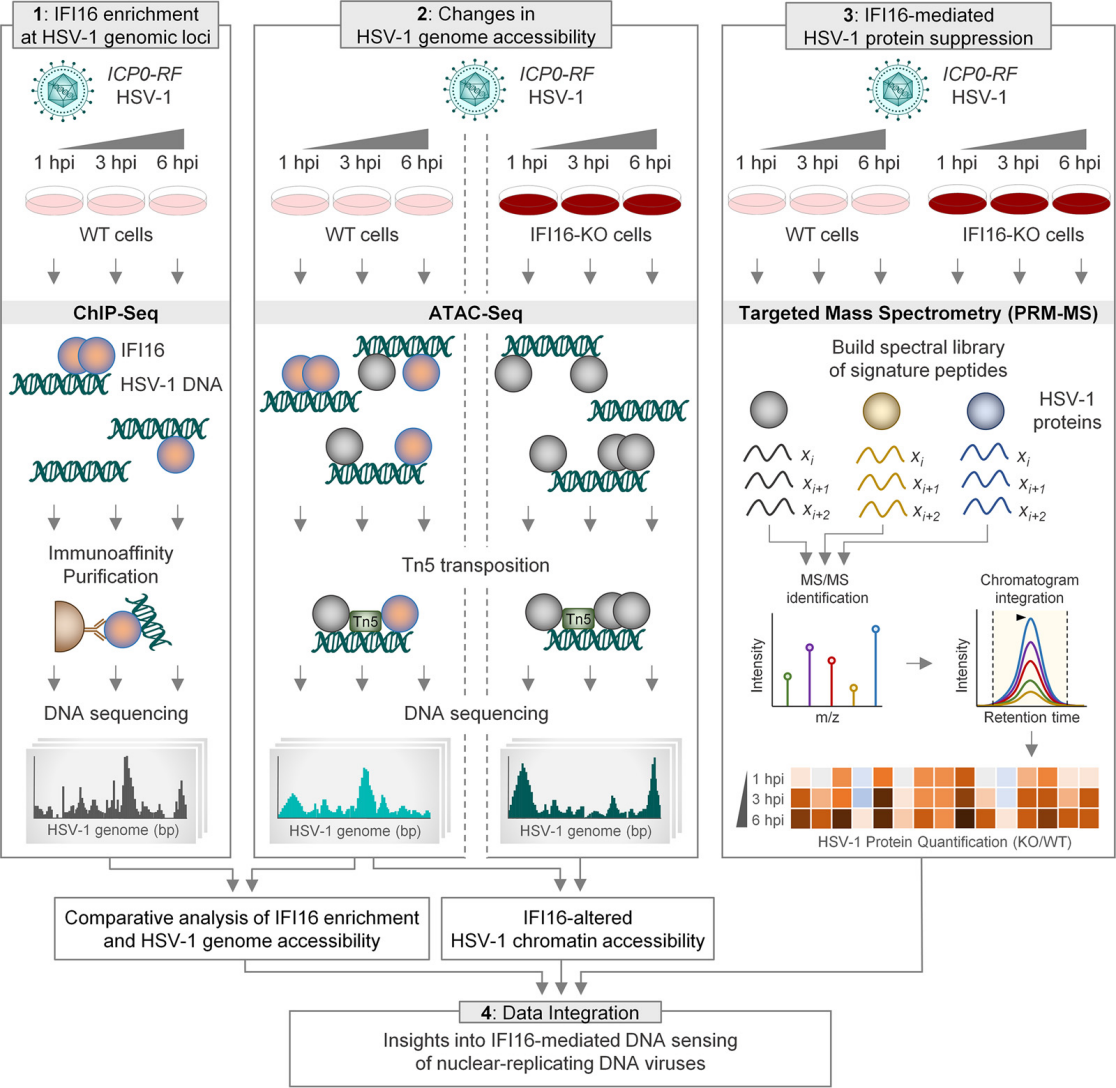
Multiomics platform to define IFI16 binding and suppression of herpesviral genomes. Omics techniques were integrated to facilitate investigation into IFI16 binding to HSV-1 DNA and IFI16-mediated suppression of viral genes. Immunoaffinity purification of IFI16-DNA complexes followed by Illumina sequencing (IFI16 ChIP-seq) was used to map interactions between IFI16 and HSV-1 DNA. Dynamics of the HSV-1 genome accessibility during infection were investigated using ATAC-seq in WT and IFI16-KO cells. A targeted MS (parallel reaction monitoring [PRM]-MS) assay monitoring HSV-1 protein abundances was used to measure differences in viral protein expression in WT and IFI16-KO cells.

The most widely accepted hypothesis indicates that IFI16 binds dsDNA in a sequence-independent manner (17, 25), although this remains to be sufficiently demonstrated in infected cells. However, several factors, including protein-protein interactions and accessibility owing to 3-dimensional DNA structures, could dictate particular regions of IFI16 enrichment on the HSV-1 genome. For example, is IFI16 directed to specific genes, such as immediate early genes, to optimally suppress viral infection? Does IFI16 have sequence binding preferences either within these genes or in upstream promoter regions? To gain a better understanding of the antiviral response nucleated by IFI16 during virus infection, we used a multiomics approach to map IFI16 binding to viral DNA and determine its impact on both viral genome accessibility and viral protein production during HSV-1 infection (Fig. 1).

**FIG 2:**
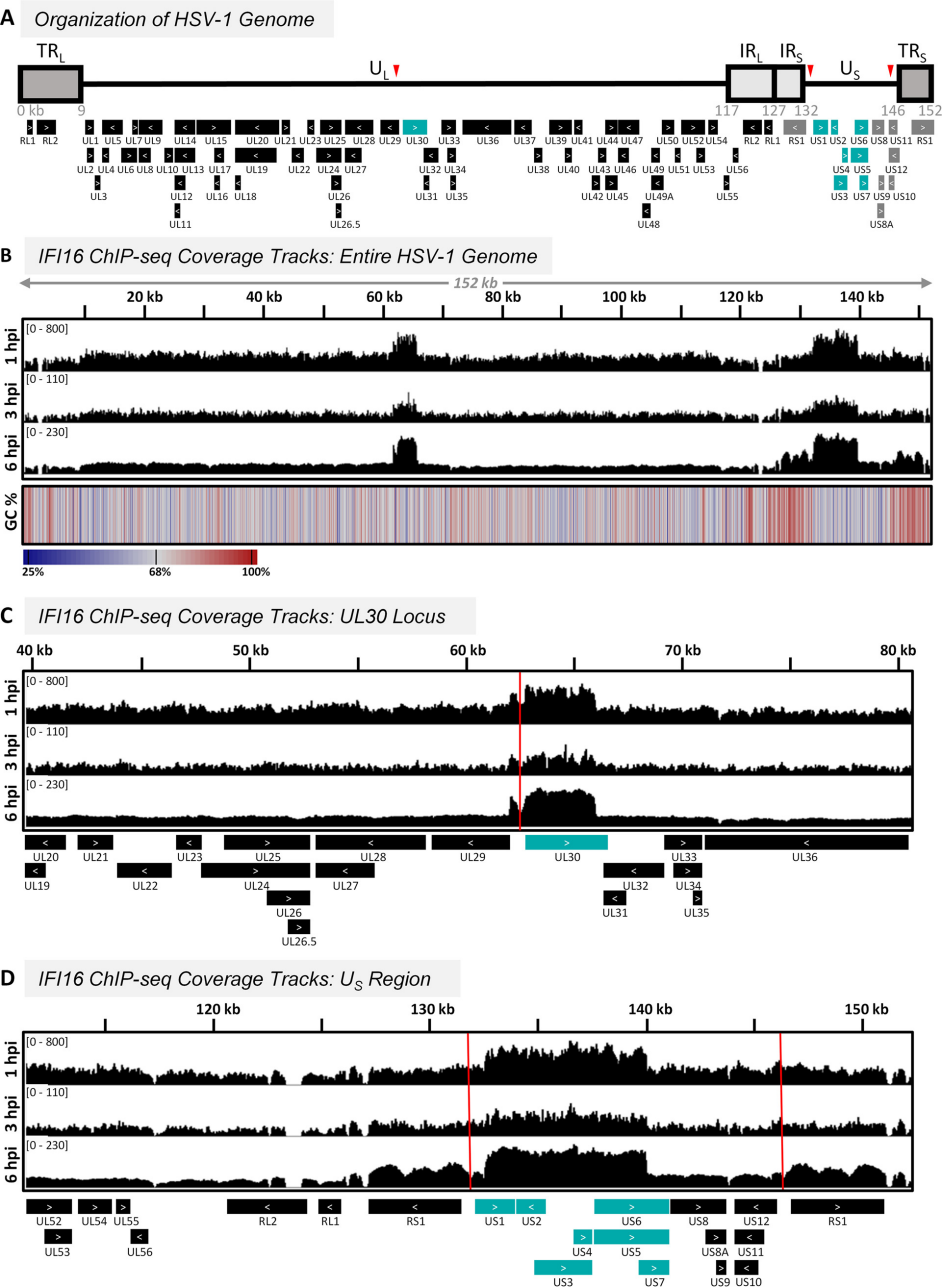
IFI16 indiscriminately binds to the HSV-1 genome and is enriched at the genes UL30 and US1–US7. (A) Schematic of the HSV-1 genome displaying the unique long (U_L_) and unique short (U_S_) regions, terminal repeats (TR_L_ and TR_S_), and inverted repeats (IR_L_ and IR_S_). Genes are shown as boxes, with inner white arrows indicating gene orientation. Genes that are highlighted in teal and gray indicate regions of interest that are discussed below (teal, ChIP-seq; gray, ATAC-seq). Red triangles mark locations of viral origins of replication. (B to D) IFI16 ChIP-seq was performed in HFF-1 cells following infection with *ICP0-RF* HSV-1, and samples were harvested at the indicated times postinfection (MOI = 10). Data were first normalized for sequencing depth and viral genome number using input sample ChIP-seq reads. Coverage tracks are displayed as the difference in average reads recovered from IFI16 and IgG immunoaffinity purifications (IFI16 − IgG). Scales representing normalized read counts are in brackets. GC percentage in panel B was measured as the average in 50-bp bins, and the mean GC content of the genome was 68%. Vertical red lines in panels C and D indicate viral origins of replication. Two biological replications were used for 1 hpi, and three biological replications were used for 3 and 6 hpi (50-bp bins).

First, we sought to map the interaction between IFI16 molecules and the HSV-1 genome. Spanning about 152 kbp in length and containing at least 80 open reading frames, the HSV-1 genome is among the largest of known human viral pathogens. The genome is organized as unique long (U_L_) and unique short (U_S_) segments flanked by inverted repeats (Fig. 2A). Using ChIP-seq, we probed the interaction between endogenous IFI16 and the HSV-1 genome during infection (Fig. S1). We collected IFI16 DNA samples from human fibroblasts during early stages of HSV-1 infection at 1, 3, and 6 h postinfection (hpi). We chose to infect fibroblasts due to the multitude of studies demonstrating anti-HSV-1 functions for IFI16 in this cell type, including observations of inducing cytokine expression, suppressing viral protein production, and repressing virion progeny production (2, 4, 8, 19–21, 28–33). We focused on early infection time points, up to 6 hpi, when we and others have observed IFI16 binding to incoming vDNA at the nuclear periphery (9, 20, 21, 28, 34–37). This binding event involves the relocalization of IFI16 into asymmetric nuclear peripheral puncta coincident with the early formation of vDNA complexes for replication. During these first 6 h of infection, the virus rapidly progresses through a temporal cascade of immediate early (IE), early (E), and late (L) gene expression before beginning vDNA replication (38). After 6 hpi, nascent vDNA begins to be packaged into capsids within the nucleus, thereby becoming inaccessible to IFI16 binding. Wild-type (WT) HSV-1 expresses the IE protein ICP0 to aid in immune evasion by promoting the degradation of IFI16 and other host factors before 6 hpi (9, 21). As such, we utilized an *ICP0-RF* mutant virus lacking the E3 ubiquitin ligase activity of ICP0 (39), which does not cause IFI16 degradation (21, 28, 30). We noted that the *ICP0-RF* mutant lacks the introns located in the two WT copies of the ICP0 gene RL2. As such, when mapping our sequencing data to the HSV-1 strain 17 genome (NCBI reference sequence NC_001806.2), there were short gaps of missing values in the two RL2 genes where the introns are located in the WT genome.

10.1128/msystems.00198-22.1 FIG S1 IFI16 immunoaffinity isolation for use in ChIP-seq and IFI16 binding within HSV-1 gene bodies. (A) Endogenous antibody efficiently isolates IFI16 protein; 1% input, 2.5% elution, 1% flowthrough. (B) IFI16-KO HFF-1 cells were generated to confirm specificity of the antibody used for IFI16 IP. The efficiency of IFI16 knockout was determined by measuring *IFI16* mRNA levels via RT-qPCR. mRNA levels were normalized to *β-actin*. Values are means and SD from three technical replicates. (C) The IFI16 antibody used for IP is specific to IFI16. HFF-1 Ctrl and IFI16-KO cells were probed for IFI16. Integrated densities of IFI16 bands were normalized by the integrated densities of tubulin bands, and the ratio to Ctrl is reported. (D) Line graphs representing the average IFI16 ChIP-seq signal in 50-bp bins in all HSV-1 open reading frames, scaled to 2 kbp. Download FIG S1, TIF file, 2.8 MB. Copyright © 2022 Howard et al. 2022 Howard et al. https://creativecommons.org/licenses/by/4.0/ This content is distributed under the terms of the Creative Commons Attribution 4.0 International license.

In agreement with previous findings that IFI16 binds to dsDNA in a sequence-independent manner (17, 24), IFI16 interacted with the entire HSV-1 genome at each time postinfection (Fig. 2B). In previous protein-protein interaction studies, we have shown that IFI16 interacts with RNA polymerase II and several host and viral transcription factors during HSV-1 infection (4, 9, 20, 30). As such, we considered if IFI16 binding was enriched upstream of HSV-1 genes in the promoter regions. Analysis of the average IFI16 ChIP signal from each HSV-1 gene, including 1 kbp up- and downstream, demonstrated a slight enrichment of IFI16 at the starts of viral genes at 1 hpi and the ends of genes at 6 hpi (Fig. S1D). However, as the average signal within the genes greatly varied, we could not draw any conclusions about preferential binding of IFI16 relative to HSV-1 gene bodies.

We observed two broad loci of IFI16 enrichment that include the genes UL30 (Fig. 2C) and US1–US7 (Fig. 2D). The products of the UL30 and US1 genes are essential for HSV-1 replication. UL30 encodes the catalytic subunit of the HSV-1 DNA polymerase (40). In turn, the US1 gene produces the IE protein ICP22, a regulatory protein which is required for expression of several late genes and which acts by modulating the phosphorylation state of the cellular RNA polymerase II (41). The US2, US3, and US4–US7 genes code for an envelope-associated protein, viral kinase, and glycoproteins, respectively. We asked whether IFI16 specifically targets these genes because of local differences in GC content. At UL30, the GC percentage within the region of IFI16 enrichment is similar to that of the adjacent genes (Fig. 2B). In contrast, the GC content of IR_S_ is much higher than that of the U_S_ region, and we observed a stark shift in IFI16 binding at this location, suggesting anticorrelation between GC percentage and IFI16 enrichment. However, GC content then remains constant throughout the U_S_ region, while IFI16 binding sharply decreases before the start of US8. These trends support the notion that the amount of IFI16 binding is not primarily driven by GC content but rather that other factors are involved.

Interestingly, both regions of IFI16 enrichment are adjacent to two *cis*-acting elements that are crucial for HSV-1 replication—the origin of replication palindromes OriL within the U_L_ region and the two OriS sequences flanking the U_S_ region (marked by red triangles in Fig. 2A and vertical red lines in Fig. 2C and D). We found that IFI16 is prominently excluded from OriL at all times postinfection and from both OriS sites at 6 hpi. Differences in IFI16 interaction could be explained by disparities in the structures of these sites. OriL is a 144-bp perfect palindrome, while the OriS sites are 45-bp imperfect palindromes (42). Further, there is evidence for functional differences between OriL and OriS wherein OriL contributes more than OriS to replication and pathogenesis *in vivo* (43). IFI16 oligomerizes upon binding naked dsDNA (25, 26), and these oligomeric structures have been shown to transition from puncta to filaments following vDNA replication (32). Thus, we hypothesize that the proximity of IFI16 enrichment to the viral origins of replication is, in part, driven by vDNA replication and concomitant changes in the local chromatin landscape. Altogether, our results demonstrate in an unbiased manner that IFI16 binds to the HSV-1 genome irrespective of DNA sequence, while also displaying enrichment at the UL30 and US1–US7 genes.

### IFI16 ChIP-seq enrichment corresponds to the most accessible regions of vDNA.

**FIG 3:**
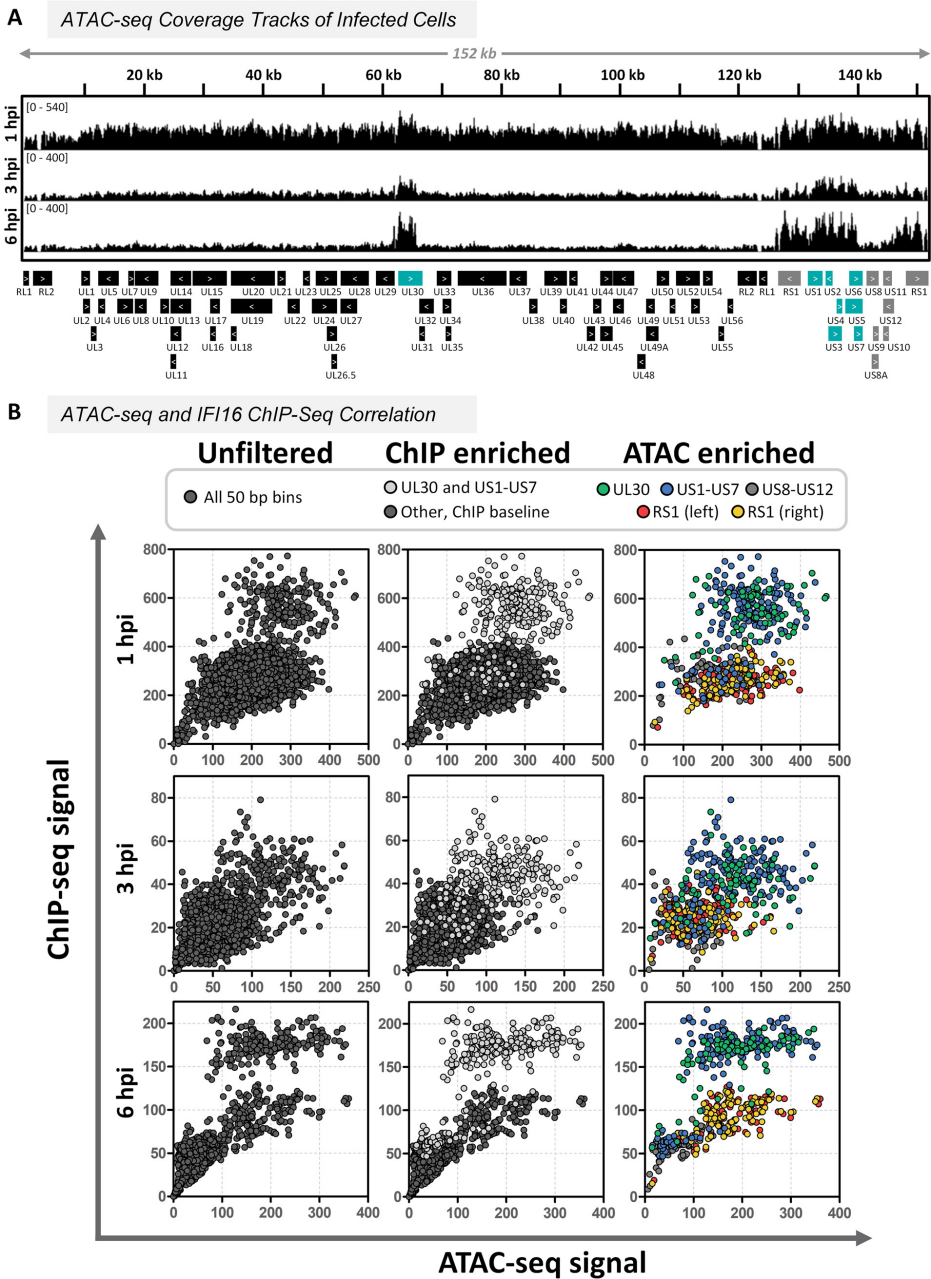
HSV-1 genome transposase accessibility correlates with IFI16 binding. (A) ATAC-seq was performed following infection with *ICP0-RF* HSV-1 and Tn*5* transposase treatment at the indicated times postinfection in control HFF-1 cells (MOI = 10). Data were normalized by sequencing depth and viral genome numbers (quantified via qPCR). Coverage tracks represent the average of three biological replicates at 1 and 3 hpi and two biological replicates for 6 hpi (50-bp bins). Scales representing normalized read counts are in brackets. (B) Scatterplots showing correlation between average ATAC-seq and average IFI16 ChIP-seq signal within each 50-bp bin. Left column, no filter; center column, bins within the IFI16 ChIP-seq-enriched genes UL30 and US1–US7 (including intergenic regions) are highlighted in light gray; right column, bins within the ATAC-seq-enriched genes are highlighted and baseline data points are excluded. Regions were determined using sequencing data at 6 hpi. UL30, 62,100 to 66,000 bp; RS1 (left), 127,100 to 131,500 bp; US1–US7, 132,000 to 141,100 bp; US8–US12, 141,100 to 146,700 bp; RS1 (right), 146,700 to 151,100 bp.

To investigate how dsDNA structure governs interactions with IFI16 during infection, we sought to identify patterns in vDNA chromatinization at early times postinfection with *ICP0-RF* HSV-1 in control fibroblasts. To this end, we performed the assay for transposase-accessible chromatin coupled with sequencing (ATAC-seq) to directly assess the vDNA chromatin landscape. This technique utilizes a hyperactive Tn*5* transposase that cuts naked DNA and tags the free ends for subsequent Illumina sequencing, in order to profile the chromatin state of dsDNA in an unbiased fashion (44). To ensure that data could be compared between samples collected at different times postinfection, reads were normalized by relative HSV-1 genome quantification in each sample (Fig. S2). At 1 hpi, we observed that the entire genome was highly transposase accessible (Fig. 3A). By 3 and 6 hpi, sequencing reads from several regions were overrepresented, indicating that these loci had become more accessible. Specifically, we observed an elevated ATAC-seq signal at 62 to 66 kbp (covering UL30), 127 to 141 kbp (including US1–US7), and 147 to 151 kbp. Interestingly, the former two loci correspond to the regions of IFI16 enrichment established by ChIP-seq (Fig. 2). Beyond the context of IFI16, our data are consistent with data from recent ATAC-seq experiments that reveal uniform read counts along the length of the HSV-1 genome, as well as DNA fragment size profiles lacking nucleosomal banding patterns (Fig. S2B) (45, 46). Collectively, our data are in line with previous findings and provide support to the prevailing hypothesis that the HSV-1 genome is loaded with histones and other proteins shortly after entering the nucleus (47, 48). However, unlike host dsDNA, the HSV-1 genome does not appear to be organized into regularly repeating nucleosomes.

10.1128/msystems.00198-22.2 FIG S2 HSV-1 genome quantification for ATAC-seq normalization. (A) Cell samples were split from Ctrl and IFI16-KO cell samples destined for ATAC-seq analysis following infection with *ICP0-RF* HSV-1 at 1, 3, and 6 hpi (MOI = 10). HSV-1 genomes were quantified by measuring UL5 and UL30 genomic DNA levels via RT-qPCR and then normalizing them to one IFI16-KO replicate. Values are the means and SD from three technical replicates. (B) HSV-1 genome fragment sizes in Ctrl fibroblasts following Tn*5* transposition. The line graph shows the means for all control cell samples. Download FIG S2, TIF file, 1.1 MB. Copyright © 2022 Howard et al. 2022 Howard et al. https://creativecommons.org/licenses/by/4.0/ This content is distributed under the terms of the Creative Commons Attribution 4.0 International license.

To determine if there was correlation between local increased transposase accessibility and loci of IFI16 binding, we plotted IFI16 ChIP-seq signal versus the ATAC-seq signal in 50-bp bins (Fig. 3B). Examining all data in each time point (Fig. 3B, left column), we observed distinct populations with elevated signal from both ChIP-seq and ATAC-seq. We then highlighted the regions of elevated IFI16 binding (UL30 and US1–US7, shown in light gray in the center column) to determine if the elevated ChIP-seq signal matched the ATAC-seq signal. Indeed, the amount of IFI16 binding to the HSV-1 genome correlated with increased transposase-accessibility of the HSV-1 genome. These data thus support our hypothesis that disparities in the vDNA 3-dimensional structure drive higher IFI16 binding and cooperative assembly. To further examine the relationship between ATAC-seq signal and IFI16 enrichment, we then removed all bins with baseline ATAC-seq signal (retaining those contiguous with elevated signal). This winnowed our analysis to five distinct regions: UL30, US1–US7, US8–US12, and the two RS1 copies. Interestingly, the numbers of ChIP-seq reads in UL30 and US1–US7 were generally higher than in US8–US12 and RS1 when compared to similar ATAC-seq signals. This could imply that IFI16 enrichment is not exclusively driven by cooperative assembly on naked vDNA but rather that other viral or host factors could promote the accumulation of IFI16 molecules at specific loci.

### IFI16 globally reduces HSV-1 genome accessibility.

We next asked how the expression of IFI16 affects the chromatin landscape of HSV-1. During HSV-1 infection, IFI16 suppresses viral gene expression, and several studies have suggested a mechanism in which IFI16 promotes heterochromatinization of vDNA during HSV-1 infection (28, 29). However, a more comprehensive assay is required to elucidate how IFI16 binding both globally and locally affects the structure of the entire HSV-1 genome. We generated control (Ctrl) and IFI16 knockout (IFI16-KO) fibroblasts via CRISPR/Cas9 technology (Fig. S1B) and employed ATAC-seq to measure differences in genome accessibility driven by IFI16. Sequencing data were again normalized by viral genome quantification to accurately compare between times postinfection (Fig. S2).

**FIG 4:**
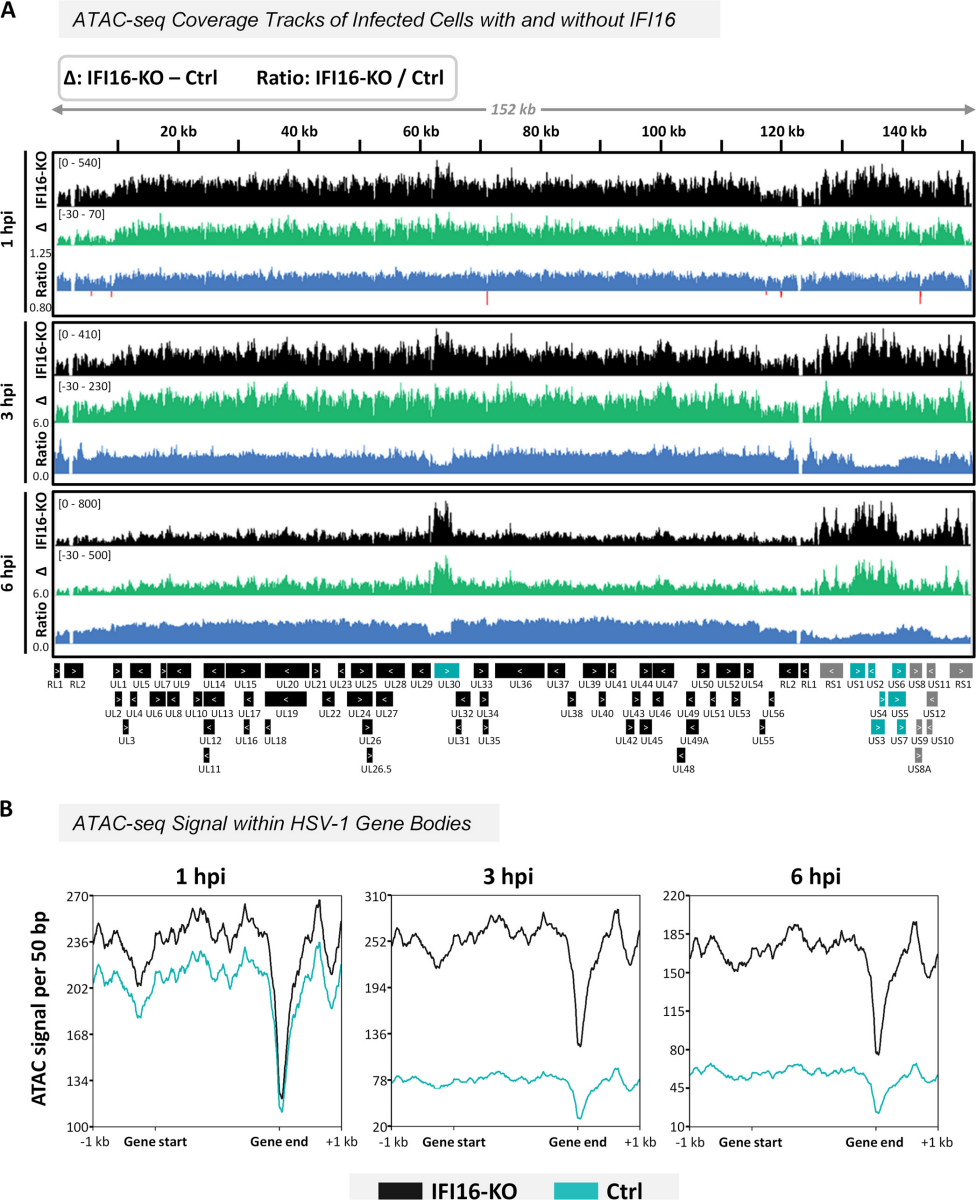
IFI16 reduces transposase accessibility of the HSV-1 genome throughout infection. (A) ATAC-seq data from *ICP0-RF*-infected IFI16-KO HFF-1 cells (1, 3, and 6 hpi; MOI of 10) were normalized by sequencing depth and viral genome numbers (quantified via qPCR). Coverage tracks at each time postinfection represent the average of biological replicates from IFI16-KO cells, the difference between IFI16-KO and Ctrl cells (data shown in Fig. 2; Δ, IFI16-KO minus control), and the ratio between IFI16-KO and control cells (IFI16-KO/Ctrl). The baseline for the ratio coverage tracks was set at 1.0 in each graph, and all values below 1.0 are red. Three biological replicates were used for 1 and 3 hpi, and two biological replicates were used for 6 hpi (50-bp bins). Scales representing normalized read counts are in brackets. (B) Line graphs representing the average ATAC-seq signal from IFI16-KO (black) and control (teal) cells in all HSV-1 open reading frames, scaled to 2 kbp.

Comparing the ATAC-seq signal between the Ctrl and IFI16-KO samples, we obtained more reads across the entire HSV-1 genome in IFI16-KO cells at all times postinfection (Fig. 4A). This increase became more dramatic as infection proceeded, as evidenced by the increases at 3 and 6 hpi in the coverage tracks representing the differences and ratios between IFI16-KO and Ctrl cells. Expression of IFI16 thus globally reduces vDNA transposase accessibility, regardless of IFI16 enrichment at specific loci. This process occurs progressively in the hours following entry of vDNA into the nucleus. Additionally, we investigated whether IFI16 specifically targeted the upstream promoter regions of HSV-1 genes for chromatinization. As is typical for ATAC-seq, the number of reads for viral genes in both Ctrl and IFI16-KO cells is highest in the promoter regions and within the gene bodies before dramatically dropping at the transcription end sites (Fig. 4B). Interestingly, at 1 hpi, the promoters and gene bodies are more accessible in the IFI16-KO cells, but the ends of the genes yielded similar signals in both Ctrl and IFI16-KO. In comparison, at 3 and 6 hpi, lack of IFI16 expression led to increased ATAC-seq signal without any positional dependence relative to the gene bodies.

We also found that, at 3 and 6 hpi, the increase in genome accessibility associated with IFI16-KO was less dramatic at UL30 and US1–US7 compared to regions of low IFI16 enrichment (Fig. 4A). This is clearly represented by the depressions in the coverage tracks for the IFI16-KO/Ctrl ratio at these times postinfection. Given the importance of these regions for vDNA replication, our results suggest that locus-specific immune evasion mechanisms exist to protect the DNA replication process.

### IFI16 globally represses HSV-1 protein expression.

Having mapped the binding of IFI16 to the HSV-1 genome and determined that expression of IFI16 broadly decreases vDNA accessibility, we next aimed to establish whether this results in a global suppression of HSV-1 protein expression or, alternatively, whether our observed IFI16 enrichment at UL30 and US1–US7 leads to greater suppression of these genes than others. To address this, we designed a targeted mass spectrometry (MS) assay to concurrently detect and quantify HSV-1 proteins in Ctrl and IFI16-KO cells at 1, 3, and 6 hpi. This MS assay, based on parallel reaction monitoring (PRM), surveyed 59 HSV-1 proteins (~80% of the HSV-1 canonical proteome) comprising every temporal expression class (IE, E, and L).

**FIG 5:**
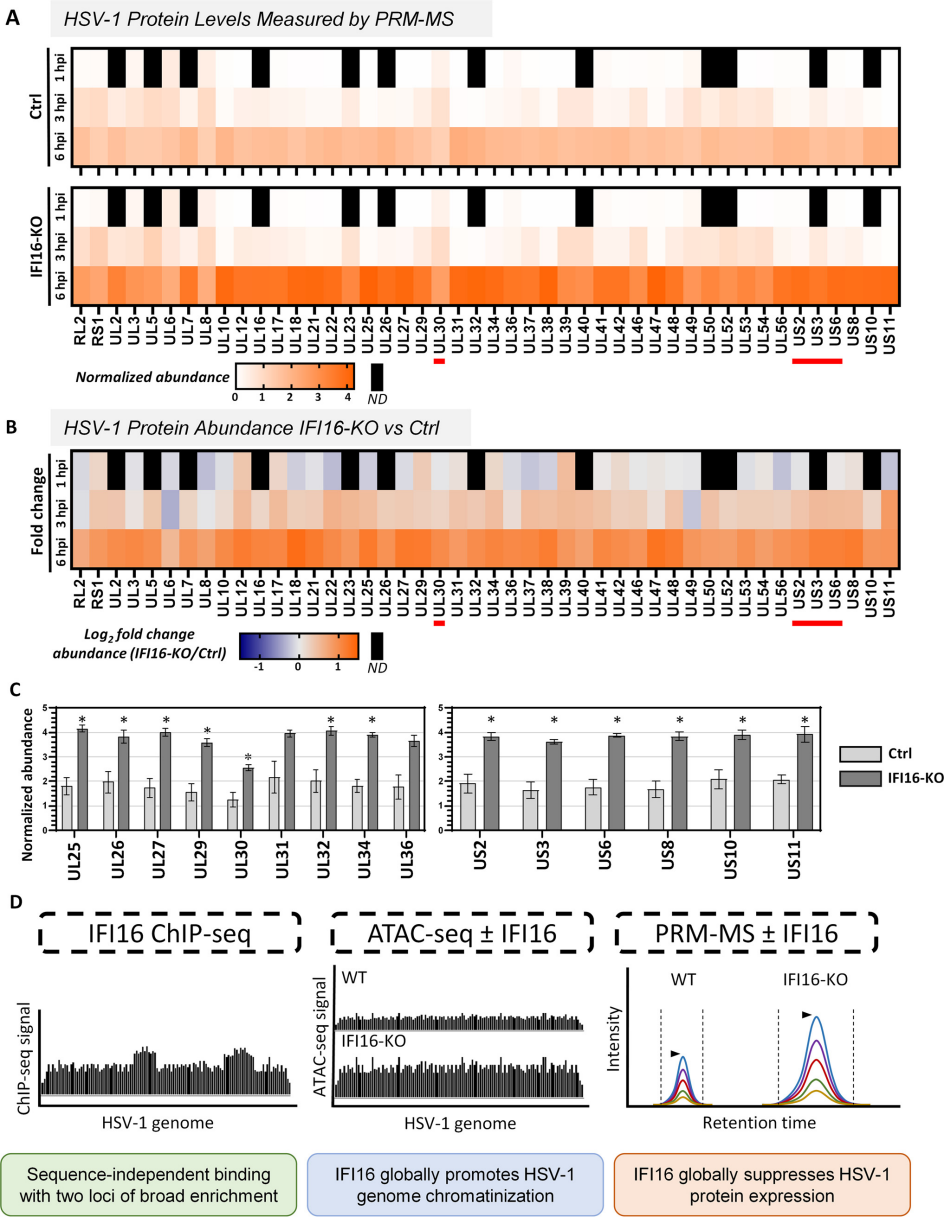
Expression of IFI16 globally suppresses HSV-1 protein levels. Targeted PRM-MS was used to quantify HSV-1 protein levels in Ctrl and IFI16-KO cells following infection with *ICP0-RF* HSV-1 at 1, 3, and 6 hpi (MOI = 5). Three biological replicates. (A) Heat maps represent the normalized abundance of HSV-1 proteins in Ctrl and IFI16-KO cells, quantified from two or more precursor ions. (B) Log_2_ fold change between IFI16-KO and Ctrl cells. ND, not detected. Horizontal red lines in panels A and B indicate regions of IFI16 enrichment. (C) Bar graphs displaying the protein abundances of the genes surrounding UL30 and in the U_S_ region. Error bars represent the standard errors of the means. Values that are significantly different between IFI16-KO and Ctrl samples (Student's *t* test, *P* < 0.05) are indicated by an asterisk. (D) Key insights into the IFI16 antiviral mechanism revealed by multiomics approach of ChIP-seq (left), ATAC-seq (center), and targeted mass spectrometry (right).

In both Ctrl and IFI16-KO cells, we observed an accumulation of viral proteins as infection progressed, in agreement with an adequate progression of infection (Fig. 5A; Fig. S3; Table S1). At the earliest stages of infection, only five viral proteins are produced: the IE proteins ICP0, ICP4, ICP22, ICP27, and ICP47, encoded by the genes RL2, RS1, UL1, UL54, and US12, respectively. These proteins function to both dampen the cellular immune response and initiate the expression of E viral proteins. At 1 hpi, we detected ICP0 (RL2), ICP4 (RS1), and ICP27 (UL54) and observed no significant differences between Ctrl and IFI16-KO cells (Fig. 5B and Fig. S4). Although they were not actively expressed at this early time during infection, we also detected 40 other viral proteins at 1 hpi, most of which are expected to have entered the cell as integral virion components. We did not find significant differences in the abundance of these viral proteins by 1 hpi. As IFI16 has been demonstrated to suppress the expression of new viral proteins (28), rather than promote the degradation of existing virion components, our data support this antiviral function. At 3 and 6 hpi, viral protein levels continued to increase in both Ctrl and IFI16-KO cells. Overall, the rate at which viral protein abundances increased during infection was higher in IFI16-KO cells, indicating that IFI16 begins to globally restrict HSV-1 protein expression at or before 3 hpi. This trend continued through 6 hpi, when all viral proteins were more abundant in IFI16-KO than in Ctrl cells, with an observed 2-fold difference for most proteins. Additionally, IFI16-KO led to a reduction in cytokine induction at 6 hpi (Fig. S5), consistent with previous findings (9) and the IFI16-mediated reduction of viral protein levels observed in this PRM assay.

10.1128/msystems.00198-22.3 FIG S3 Abundances of all HSV-1 proteins monitored by PRM. Targeted PRM-MS was used to quantify HSV-1 protein levels in Ctrl and IFI16-KO cells following infection with *ICP0-RF* HSV-1 at 1, 3, and 6 hpi (MOI = 5). Three biological replicates were used. Proteins displayed include all viral proteins monitored in which one or more precursor ion was detected. (A) Heat maps represent the normalized abundance for HSV-1 proteins in Ctrl and IFI16-KO cells. (B) Log_2_ fold change between IFI16-KO and Ctrl cells. ND, not detected. Horizontal red lines in panels A and B indicate regions of IFI16 enrichment. Download FIG S3, TIF file, 1.5 MB. Copyright © 2022 Howard et al. 2022 Howard et al. https://creativecommons.org/licenses/by/4.0/ This content is distributed under the terms of the Creative Commons Attribution 4.0 International license.

10.1128/msystems.00198-22.4 FIG S4 Abundances of HSV-1 proteins in Ctrl and IFI16-KO cells, organized by temporal gene class. Targeted PRM-MS was used to quantify HSV-1 protein levels in Ctrl and IFI16-KO cells following infection with *ICP0-RF* HSV-1 at 1, 3, and 6 hpi (MOI = 5). Three biological replicates were used. Proteins are organized based on temporal gene class, and only proteins quantified from two or more precursors ions are displayed. (A) Heat maps represent the normalized abundance for HSV-1 proteins in Ctrl and IFI16-KO cells. (B) Log_2_ fold change between IFI16-KO and Ctrl cells. ND, not detected; IE, immediate early; E, early; L, late. Download FIG S4, TIF file, 2.1 MB. Copyright © 2022 Howard et al. 2022 Howard et al. https://creativecommons.org/licenses/by/4.0/ This content is distributed under the terms of the Creative Commons Attribution 4.0 International license.

10.1128/msystems.00198-22.5 FIG S5 IFI16-KO reduces cytokine expression during HSV-1 expression. (A) Ratios of cytokine mRNA levels in Ctrl and IFI16-KO fibroblasts infected with *ICP0-RF* HSV-1 (MOI of 10) at 6 hpi compared to mock-infected cells after normalization to *gapdh* levels. Values are means and standard errors of the means (*n *= 3). Statistically significant differences between Ctrl and IFI16-KO cells were determined by Student’s *t* test. **, *P* ≤ 0.005. Download FIG S5, TIF file, 0.5 MB. Copyright © 2022 Howard et al. 2022 Howard et al. https://creativecommons.org/licenses/by/4.0/ This content is distributed under the terms of the Creative Commons Attribution 4.0 International license.

10.1128/msystems.00198-22.6 TABLE S1 Abundances of peptides targeted during *ICP0-RF* HSV-1 infection (6 hpi, MOI = 5) of Ctrl and IFI16-KO fibroblasts measured by targeted MS/MS analysis via PRM. Download Table S1, TIF file, 0.1 MB. Copyright © 2022 Howard et al. 2022 Howard et al. https://creativecommons.org/licenses/by/4.0/ This content is distributed under the terms of the Creative Commons Attribution 4.0 International license.

To address if IFI16 enrichment at specific genomic loci correlated with the degree of IFI16-mediated restriction in HSV-1 proteins, we focused on the differences between Ctrl and IFI16-KO cells in the regions surrounding UL30 and US1–US7 at 6 hpi (Fig. 5C). Here, we did not observe any differences between UL30 and the products of the four flanking genes on each side. Similarly, the differences between proteins from Ctrl and IFI16-KO samples within the U_S_ region did not vary considerably between the genes at which IFI16 was enriched (US2, US3, and US6) compared to the adjacent genes (US8, US10, and US11). Altogether, these data demonstrate that IFI16-mediated suppression is globally applied across the entire genome rather than being a preferential inhibition of protein expression from the UL30 and US1–US7 loci.

## DISCUSSION

Understanding the interplay between DNA sensors and target DNA is critical for establishing the mechanisms by which these proteins initiate immune responses. This is particularly relevant for nuclear DNA sensors, which must differentiate between host and pathogen-derived DNA existing side-by-side within the nucleus. Beyond host-pathogen interactions, inadvertent binding of DNA sensors to host DNA and subsequent immune signaling could have dire consequences for cellular and tissue homeostasis. For example, aberrant expression of IFI16 has been linked to several autoimmune diseases, including systemic lupus erythematosus (SLE) and Sjögren’s syndrome (49, 50). Further, it has been suggested that oligomerization of IFI16 upon dsDNA stimulates the establishment of IFI16 as an autoantigen in target tissues of Sjögren’s syndrome (51). Thus, investigations of specific IFI16 binding loci and biochemical cues for IFI16 binding in contexts of innate immune activation, including pathogen infection, are warranted.

In this study, we applied a multiomics approach that combined high-throughput next-generation DNA sequencing techniques with our designed targeted mass spectrometry assay and performed the first mapping of the interaction between a nuclear DNA sensor and pathogenic DNA during viral infection. Previous *in vitro* studies suggested that IFI16 binds to dsDNA in a sequence-independent manner (1, 17, 25, 26). However, a detailed characterization of in what regions, and to what extent, IFI16 binds HSV-1 genomes during infection had not been performed. To this end, we utilized ChIP-seq of endogenous IFI16 during HSV-1 infection at 1, 3, and 6 hpi to probe the interaction between this nuclear DNA sensor and vDNA. Supporting *in vitro* observations of sequence-independent binding, we found that IFI16 binds to the entire HSV-1 genome, and this binding exhibited no distinct peaks dependent on any DNA sequence motifs. Rather, at all times postinfection measured, we observed two regions of broad IFI16 enrichment: the viral genes UL30 and US1–US7 (Fig. 2 and 5D). Although we found no evidence for correlation of IFI16 enrichment and vDNA GC content, these two loci share a feature in their proximity to the viral origins of replication. It is tempting to propose that IFI16 enrichment is driven by vDNA replication, either directly through changes in vDNA structure or indirectly through interactions with other proteins involved in replication of the viral genome.

During HSV-1 infection, IFI16 forms higher-order oligomeric structures critical for its antiviral functions. Our lab previously established that expression of an IFI16 mutant deficient in oligomerization capacity severely reduces IFI16-mediated cytokine induction and suppression of viral gene expression (20). Interestingly, as observed by confocal microscopy in cells, IFI16 oligomers are dynamic and form small, spherical puncta immediately following entry of the viral genome into the nucleus (9). Upon the onset of HSV-1 DNA replication, IFI16 oligomers develop into long, filamentous structures (9, 30, 32), indicating that IFI16 oligomerizes upon newly synthesized strands of vDNA. Further, given that cooperative assembly of IFI16 oligomers depends upon the availability of free DNA in both length and concentration (25), we investigated how changes in the vDNA chromatin landscape can drive IFI16 enrichment. Using ATAC-seq, we measured the accessibility of the HSV-1 genome at 1, 3, and 6 hpi. Our data demonstrated that the observed IFI16 enrichment correlates with increased vDNA accessibility at UL30 and in the U_S_ region (Fig. 3). These changes in the structure of the viral genome could be attributed either to associations with viral and host proteins or, at 6 hpi, the synthesis of nascent vDNA. For example, although the HSV-1 genome enters the nucleus as naked dsDNA (52), it quickly and dynamically associates with histones (48, 53, 54). However, histones appear to not form stable, regularly spaced nucleosome structures on the viral genome. This is supported by the lack of nucleosomal banding patterns in the vDNA fragment size profiles of previous HSV-1 ATAC-seq studies (45, 46), which we also recapitulated (Fig. S2B). Additionally, it is possible that the enhanced IFI16 ChIP-seq signal at UL30 and US1–US7, beyond the expected correlation between ChIP-seq and ATAC-seq signals (Fig. 3B), could be driven by interactions between IFI16 and other vDNA-binding proteins. Indeed, IFI16 has previously been shown to associate with many proteins that interact with the HSV-1 genome, including the viral replication proteins UL5, UL30, and UL42 and host proteins of the ND10 complex PML, SP100, and SUMO1 (30, 35). However, ChIP-seq experiments have not been performed with these proteins in the context of infection, and it remains to be seen if IFI16 enrichment patterns match those of another protein. As the chromatin state of the HSV-1 genome is dynamic and influenced by a multitude of associated proviral and antiviral cellular factors, it is possible that IFI16 contributes to restricting viral transcription through such a mechanism. Using IFI16-KO cells, we established that expression of IFI16 decreases accessibility to the entire HSV-1 genome, and this effect does not correlate with locus-specific IFI16 enrichment (Fig. 4 and 5D). Our ATAC-seq findings are supported by previous observations that IFI16 knockdown correlates with decreased interaction between vDNA and the histone H3, as well as increased euchromatin marks and decreased heterochromatin marks on vDNA (28).

There are several possible models for how IFI16 exerts these effects. First, recruitment of histones to the vDNA could be driven by IFI16, or as in the case of ATRX (55), histones already interacting with the viral genome could be stabilized by IFI16. Second, IFI16 binding could recruit other antiviral proteins to the vDNA via protein-protein interactions, thereby reducing genome accessibility. We previously demonstrated that IFI16 oligomerization is necessary for interaction with several transcription factors with functions in histone modification, including UBTF and the PAF1 complex (20). Finally, IFI16 itself could act as “chromatin” and reduce the accessibility of vDNA by sterically hindering proteins such as RNA polymerase II and the viral DNA polymerase. However, as we did not observe a correlation between local IFI16 enrichment and reduction in vDNA accessibility, this seems unlikely. In fact, we observed a resistance to the IFI16-mediated reduction in viral genome accessibility at UL30 and the U_S_ region, where IFI16 is most enriched at 6 hpi (Fig. 4). Viral immune evasion factors, like ICP0, act in a manner which broadly protects viral transcription and replication by targeting cellular immune signaling proteins (56). However, it is conceivable that a mechanism exists in which an immune evasion environment is formed at these regions of the genome, and these regions become privileged from the effects of antiviral proteins like IFI16. Alternatively, as these genomic loci coincide with the origins of replication, nascent vDNA molecules could also be resistant to the effects of IFI16.

We further examined if IFI16 enrichment locally enhances suppression of HSV-1 gene expression or if, like we observed in the ATAC-seq data, expression of IFI16 globally reduces viral protein levels. Indeed, we observed a global increase in measured HSV-1 protein levels upon IFI16 knockout, indicating that IFI16 enrichment on the vDNA does not lead to targeted gene suppression (Fig. 5). However, an aspect to consider is that, given the temporal expression of HSV-1 genes, any IFI16-mediated reduction in IE protein levels would halt progression of HSV-1 gene expression, resulting in lower levels of E and L proteins. This is a possible outcome, since we found that IFI16 does indeed reduce the expression of IE proteins by 3 hpi (Fig. S4). We also expect that, beyond 6 hpi, the disparity between viral protein levels in Ctrl and IFI16-KO cells would continue to widen, as we have previously observed for up to 12 hpi by Western blotting for ICP4 (IE), ICP27 (IE), and ICP8 (E) (9). However, the broad binding of IFI16 to vDNA, paired with IFI16-mediated reduction in the accessibility of the entire viral genome, suggest that IFI16 regulates gene expression from the entire HSV-1 genome. Indeed, a previous study found that IFI16 knockout results in increased association of transcription machinery with herpesvirus genes (29).

To date, our study provides the most thorough characterization of how the expression of a nuclear DNA sensor affects the expression of a herpesvirus genome. We determined that IFI16 binds to the most accessible regions of the HSV-1 genome. Simultaneously, IFI16 globally reduces vDNA accessibility and expression of all viral proteins comprising each temporal gene class. The results of our study provide insight into the functional requirement of IFI16 binding to HSV-1 genomes for the transcriptional and epigenetic restriction of infection. Previous investigations have demonstrated the utility of sequencing technologies for studying viral infections. For example, ChIP- and ATAC-seq have been used to examine how viral transcription and protein-vDNA interactions are regulated by the HSV-1 IE protein ICP4 (45) and the host chromatin modulator ATRX (46). Our study can serve as a systems-level model that integrates genomics with proteomics to evaluate nuclear DNA sensor interactions with viral genomes and downstream antiviral outcomes. Thus, future investigations can determine if IFI16 enrichment at specific viral genomic loci represents a broad mechanism dictated by local genome accessibility and if active innate immunity prior to an acute viral infection or reactivation from latency influences IFI16 enrichment.

## MATERIALS AND METHODS

### Antibodies.

Antibodies used for immunoblotting and immunoaffinity purifications were anti-IFI16 (sc-8023; Santa Cruz Biotechnology), anti-tubulin (T6199; Sigma-Aldrich), and mouse IgG (MP Biomedicals).

### Cell culture.

Primary human foreskin fibroblast-1 cells (HFF-1s) and U2OS cells were cultured in Dulbecco’s modified Eagle’s medium (DMEM) (Sigma-Aldrich) supplemented with 10% (vol/vol) fetal bovine serum (FBS) (Gemini Bio) and 1% (vol/vol) penicillin/streptomycin (Gibco) at 37°C in 5% CO_2_. HFF-1s were obtained from ATCC (SCRC-1041).

### Viruses.

The *ICP0-RF* HSV-1 mutant virus was a gift from Bernard Roizman (University of Chicago, Chicago, IL, USA) and Saul Silverstein (Columbia University, New York, NY, USA). Virus was grown in U2OS cells and collected when 100% of cells exhibited cytopathic effect. To harvest virus, both culture supernatant and cell-associated virions were collected, and the cell-associated virus samples were sonicated and centrifuged to pellet debris. Supernatants were then combined over a 10% Ficoll cushion and purified with ultracentrifugation. Virus pellets were resuspended in MNT buffer (200 mM MES, 30 mM Tris-HCl, 100 mM NaCl [pH 7.4]) and titrated by plaque assay on U2OS cells. To infect cells, virus (or no virus for mock infection) was diluted in DMEM containing 2% (vol/vol) FBS and incubated on cells at the indicated multiplicity of infection (MOI) for 1 h at 37°C and 5% CO_2_ with gentle rocking every 15 min to allow virus adsorption. Cells were washed once with phosphate-buffered saline (PBS), overlaid with complete medium described above, and incubated for the indicated lengths of time.

### Construction of stable cell lines.

IFI16-KO and control HFF-1 cell lines were produced using TrueCut Cas9 protein V2 (Thermo Fisher Scientific) and CRISPRMAX Cas9 transfection reagent (Thermo Fisher Scientific) with TrueGuide synthetic RNA (negative control, nontargeting 1; IFI16, CRISPR1100935_SGM; Thermo Fisher Scientific) according to the manufacturer’s instructions.

### Immunoblotting.

Samples were collected for Western blotting in Laemmli buffer (62.5 mM Tris-HCl [pH 6.8], 2% SDS [wt/vol], 10% glycerol [vol/vol], 0.02% bromophenol blue [wt/vol]) with 100 mM dithiothreitol (DTT) and boiled at 95°C for 5 min. All samples were resolved by 10% SDS-PAGE. Proteins were transferred to polyvinylidene difluoride (PVDF) membranes and blocked in 5% milk in Tris-buffered saline with (TBS) at room temperature for 1 h. Membranes were incubated with primary antibody in blocking solution with 0.2% (vol/vol) Tween 20 for 1 h at room temperature. Proteins were then visualized using either horseradish peroxidase-conjugated secondary antibodies and standard enhanced chemiluminescence detection or fluorescently conjugated secondary antibodies followed by visualization using an Odyssey CLx imager (LI-COR Biosciences).

### cDNA generation, genomic DNA purification, and quantitative PCR.

RNA extraction was performed using the RNeasy kit (Qiagen) according to the manufacturer’s instructions. DNA was digested using DNase I, and cDNA was generated by reverse transcription-PCR (RT-PCR) using the Superscript IV first-strand cDNA kit (Thermo Fisher Scientific) according to the manufacturer’s instructions. Genomic DNA was purified from cell lysates using phenol-chloroform/isoamyl alcohol (25:24:1, pH 6.7/8.0) (Thermo Fisher Scientific) according to the manufacturer’s instructions. For quantitative PCR, amplification was performed using Power SYBR green PCR master mix (Life Technologies, Inc.) and gene-specific primers with a Viia7 thermocycler (Thermo Fisher Scientific). Analysis was performed by direct comparison of cycle threshold (*C_T_*) values, and relative quantification of gene expression was assessed by the ΔΔ*C_T_* method using either *β-actin* or *gapdh* as the reference gene.

### ChIP sequencing.

ChIP-seq was performed with two or three biological replicates. At the indicated times postinfection, 4e6 cells per biological replicate were treated with 1% paraformaldehyde in complete medium for 7 min at 37°C. Cross-linking was then quenched by adding cold glycine to a final concentration of 125 mM and rocking for 5 min at room temperature. Cells were then washed twice with PBS, scraped into PBS containing 1× Halt protease and phosphatase inhibitor cocktail (P/PhIC; Thermo Fisher Scientific), and pelleted at 4°C. Cells were lysed in lysis buffer 1 (50 mM HEPES-KOH, 140 mM NaCl, 1 mM EDTA, 10% glycerol, 0.5% NP-40, 0.25% Triton X-100, and 1:100 P/PhIC), and nuclei were pelleted by centrifugation at 1,350 × *g* for 10 min at 4°C. Nuclei were then washed with lysis buffer 2 (10 mM Tris-HCl [pH 8.0], 200 mM NaCl, 1 mM EDTA, 0.5 mM EGTA, and 1:100 P/PhIC), spun down again, and resuspended in lysis buffer 3 (10 mM Tris-HCl [pH 8.0], 100 mM NaCl, 1 mM EDTA, 0.5 mM EGTA, 0.1% sodium deoxycholate, 0.5% *N*-lauroylsarcosine, 1:100 P/PhIC). Samples were then transferred to 1 mL AFA fiber tubes (Covaris), and DNA was sheared via sonication for 13 min with an LE220 focused ultrasonicator (Covaris) with the following parameters: peak incident power (PIP), 420; duty factor, 30%; cycles per burst (CPB), 200; temperature, 6°C (minimum/maximum = 3/9°C). Samples were then brought to 1% Triton X-100 and centrifuged at 20,000 × *g*, for 10 min at 4°C. Five percent of the sample was saved for input, and 5% was saved to check DNA shearing on a 1% agarose gel.

Biological replicates were split evenly, and immunoaffinity purification (IP) was carried out by incubating samples with 7 μg of either mouse IgG (MP Biomedicals) or anti-IFI16 (sc-8023; Santa Cruz Biotechnology) for 1 h at room temperature before adding 0.25 mg of Pierce protein A/G magnetic beads (Thermo Fisher Scientific) and incubating for another hour at room temperature while rotating end-over-end. Samples were then washed five times with radioimmunoprecipitation assay (RIPA) wash buffer (50 mM HEPES-KOH [pH 7.6], 100 mM LiCl, 1 mM EDTA, 1% NP-40, 0.7% sodium deoxycholate), washed once with TE wash buffer (10 mM Tris-HCl [pH 8.0], 100 mM EDTA, 50 mM NaCl), and eluted by heating in elution buffer (50 mM Tris-HCl [pH 8.0], 10 mM EDTA, 1% SDS) for 30 min at 65°C in a ThermoMixer (Eppendorf) with shaking at 300 rpm before spinning at 16,000 × *g* for 1 min at room temperature and transferring eluate to fresh tubes. Input and IP samples were then treated with RNase A at a final concentration of 0.4 mg/mL at 37°C, and cross-links were reversed by adding proteinase K to a final concentration of 0.4 mg/mL and heating at 57°C overnight. The input samples within each time point were pooled, and DNA was purified from all samples with the QIAquick PCR purification kit (Qiagen) and quantified with a Qubit 2.0 fluorometer (Invitrogen). Libraries were generated using the NEBNext Ultra II DNA library preparation kit (New England Biolabs) with 5 to 40 ng of DNA. Library sizes and concentrations were measured using a Bioanalyzer (Agilent Technologies) and pooled at equimolar concentrations. Fifty-base-pair paired-end sequencing was performed with an Illumina NovaSeq 6000 on one lane of an SP 100 2 × 50 bp Flowcell v1.5, performed at the Princeton University Genomics Core Facility.

### ATAC sequencing.

The Tn*5* transposition protocol was adapted from the work of Buenrostro et al. (44), following several modifications from Amanda Ackermann’s protocol protocol as in (57). Three hundred thousand cells were infected as described above and collected at the indicated times postinfection. Infected cells were pelleted and then resuspended in PBS, from which one-third (the equivalent of 100,000 cells) was saved to be used for Tn*5* transposition, while the remaining sample was split off and used for genome quantification via qPCR as described above. Cells were washed three times in ATAC wash buffer (10 mM NaCl, 3 mM MgCl_2_, 0.1% [vol/vol] NP-40, 0.1% (vol/vol) Tween 20, 0.01% [vol/vol] digitonin, 10 mM Tris-HCl [pH 7.5]) and then pelleted at 500 × *g* and 4°C for 10 min. Cells were then resuspended in 50 μL transposition mix (1× Tagment DNA buffer [Illumina], 33% [vol/vol] PBS, 0.1% [vol/vol] Tween 20, 0.01% [vol/vol] digitonin, 5% [vol/vol] Tagment DNA enzyme 1 [Illumina]) by gently pipetting up and down. Samples were then incubated at 37°C for 30 min while shaking at 1,000 rpm in a thermomixer. DNA was then isolated with a DNA Clean and Concentrator kit (Zymo Research), eluting in 10 μL elution buffer. Libraries were then generated by PCR amplification using NEBNext high-fidelity 2× PCR master mix (New England Biolabs) and quantified with a Qubit 2.0 fluorometer (Invitrogen) and a Bioanalyzer (Agilent Technologies) before pooling at equimolar concentration. Fifty-base-pair paired-end sequencing was performed with an Illumina NovaSeq 6000 on one lane of an SP 100 nt Flowcell v1.5, performed at the Princeton University Genomics Core Facility.

### ChIP-seq analysis.

Sequencing data were uploaded to the Lewis-Sigler Institute for Integrative Genomics (Princeton University, Princeton, NJ, USA) Galaxy instance. Reads were first mapped to the human genome (hg38) using Bowtie2 (58), keeping unaligned reads. These unaligned reads were then mapped to the HSV-1 strain 17 genome (NC_001806.2). Coverage bigWig files were generated using DeepTools bamCoverage (59) with 50-bp bins and manual scaling factors to normalize data. To account for differences in sequencing depth and viral genome content, normalization factors were calculated as follows: [1e6(input_cell_ + input_HSV-1_)]/[input_HSV-1_(sample_cell_ + sample_HSV-1_)]. The background signal was then subtracted by using bigwigCompare (59) to first sum the biological replicate IFI16 (IFI16_sum_) and IgG (IgG_sum_) files and then subtract IgG_sum_ from IFI16_sum_ to obtain IFI16_sum_ − IgG_sum_ within each time point. Coverage tracks were visualized using the Integrative Genomics Viewer (IGV) (60). Line plots of average IFI16 ChIP signal within HSV-1 genes were created using computeMatrix (59), with scale regions set to 2,000 bp with 1,000 bp up- and downstream of all UCSC-annotated open reading frames, and visualized by plotProfile (59).

### ATAC-seq analysis.

As described above, data were uploaded to Galaxy, and reads were aligned using Bowtie2 (58) first to the human genome (hg38) and then to the HSV-1 strain 17 genome (NC_001806.2). Virus-aligned reads were then used to generate coverage bigWig files with bamCoverage (59) with 50-bp bins and manual scaling factors to normalize data. The first step in calculating normalization factors was to use the qPCR viral genome quantification (see above) to calculate the ratio between each sample within one time point and one other sample (e.g., for 1 hpi, X/IFI16-KO_Rep1_ where X is all samples at 1 hpi, IFI16-KO, and control). This ratio was then multiplied by 1e6(sample_cell_ + sample_HSV-1_) to obtain a normalization factor which accounts for both differences in sequencing depth and viral genome content for each sample. Signals from IFI16-KO and control cells at each time point were then summed, using bigwigCompare (59), before subtraction and division were used to compare the two cell types. Data were visualized in the IGV platform (60). As described above, line plots displaying the average ATAC signal within HSV-1 genes at each time point were generated using computeMatrix (59) followed by visualization with plotProfile (59). Fragment size profiles were obtained using CollectInsertSizeMetrics from Picard Tools (https://github.com/broadinstitute/picard).

### Sample preparation for targeted MS.

Samples used for analysis were collected at the indicated times postinfection. To collect samples, cells were washed and scraped into PBS with 1:100 Halt P/PhIC (Thermo Fisher Scientific) and flash frozen as cell pellets in liquid nitrogen. Cells were thawed and lysed in lysis buffer (5% SDS, 0.5 mM EDTA, 100 mM NaCl, 100 mM Tris-HCl [pH 7.4]) and then boiled at 95°C for 3 min for two rounds with sonication in between. The Pierce bicinchoninic acid (BCA) protein assay kit (Thermo Fisher Scientific) was used to measure protein concentrations, following the manufacturer’s instructions. Fifty micrograms of protein was then reduced and alkylated with 50 mM chloroacetamide (CAM) and 25 mM TCEP [Tris(2-carboxyethyl)phosphine hydrochloride] before addition of aqueous phosphoric acid to a final concentration of ~1.2%. Digestion was then performed with 2 μg trypsin and suspension trapping columns (S-Trap; Protifi) for 1 h, following the manufacturer’s instructions. Peptides were then resuspended at 0.75 μg/μL in 1% formic acid (FA), 1% acetonitrile (ACN).

### Targeted MS acquisition and analysis.

Parallel reaction monitoring MS was used to detect and quantify viral peptides and was performed on a Q Exactive HF mass spectrometer (Thermo Fisher Scientific) coupled to an EASYSpray ion source (Thermo Fisher Scientific). A Dionex Ultimate 3000 nanoRSLC (Thermo Fisher Scientific) equipped with a 25 cm EASYSpray C_18_ column (Thermo Fisher Scientific; ES902) was used to separate peptides by reverse-phase chromatography as follows. Solvents A (0.1% formic acid) and B (90% acetonitrile, 0.1% formic acid) were utilized for a two-phase linear gradient of 2 to 22% solvent B for 45 min and 22 to 38% solvent B for 15 min at a flow rate of 250 nL/min. A single duty cycle consisted of an MS-SIM scan (400 to 2,000 *m/z* range, 15,000 resolution, 15-ms maximum injection time [MIT], 3 × 10^6^ automatic gain control [AGC] target) followed by 30 PRM scans (30,000 resolution, 60-ms MIT, 1 × 10^5^ AGC target, 0.8 *m/z* isolation window, normalized collision energy [NCE] of 27, 125 *m/z* fixed first mass), and spectrum data were recorded in profile. PRM assays were designed and analyzed using the Skyline Daily software (61). Data acquisition was controlled by scheduled inclusion lists with 20 concurrent precursors, resulting in two injections per sample. All peptide abundance values were first scaled to the mean abundances acquired in the MS1 scans of all injections before scaling by mean peak area within each precursor across all samples. GraphPad Prism (ver. 9) was used for data visualization and statistical analysis.

### Data availability.

The ChIP-seq data and the ATAC-seq data generated in this work have been deposited in the SRA database under accession number PRJNA809189. The PRM assay and raw MS files have been deposited in the Panorama repository for targeted MS assays and the ProteomeXchange Consortium repository. The data can be accessed at https://panoramaweb.org/L0COK2.url, and the accession number for the MS data is PXD031850.
