# Supplementary figures and images for: The Nuclear DNA Sensor IFI16 Indiscriminately Binds to and Diminishes Accessibility of the HSV-1 Genome to Suppress Infection
Source: mSystems. 2022 May 16;7(3):e00198-22. doi: 10.1128/msystems.00198-22 (PMC9239196; doi:10.1128/msystems.00198-22)

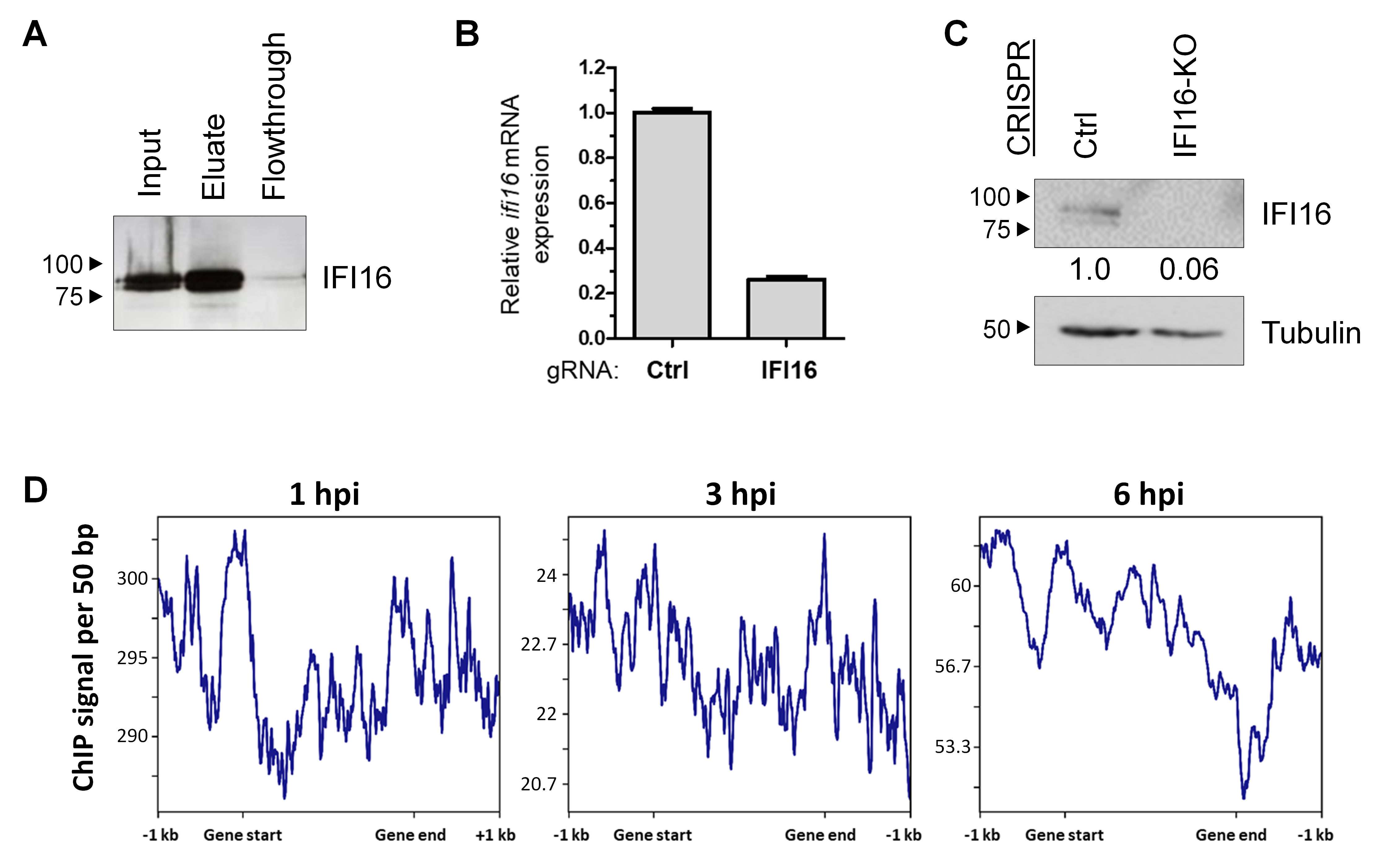

Supplement: FIG S1 [file msystems.00198-22-s0002.tif]

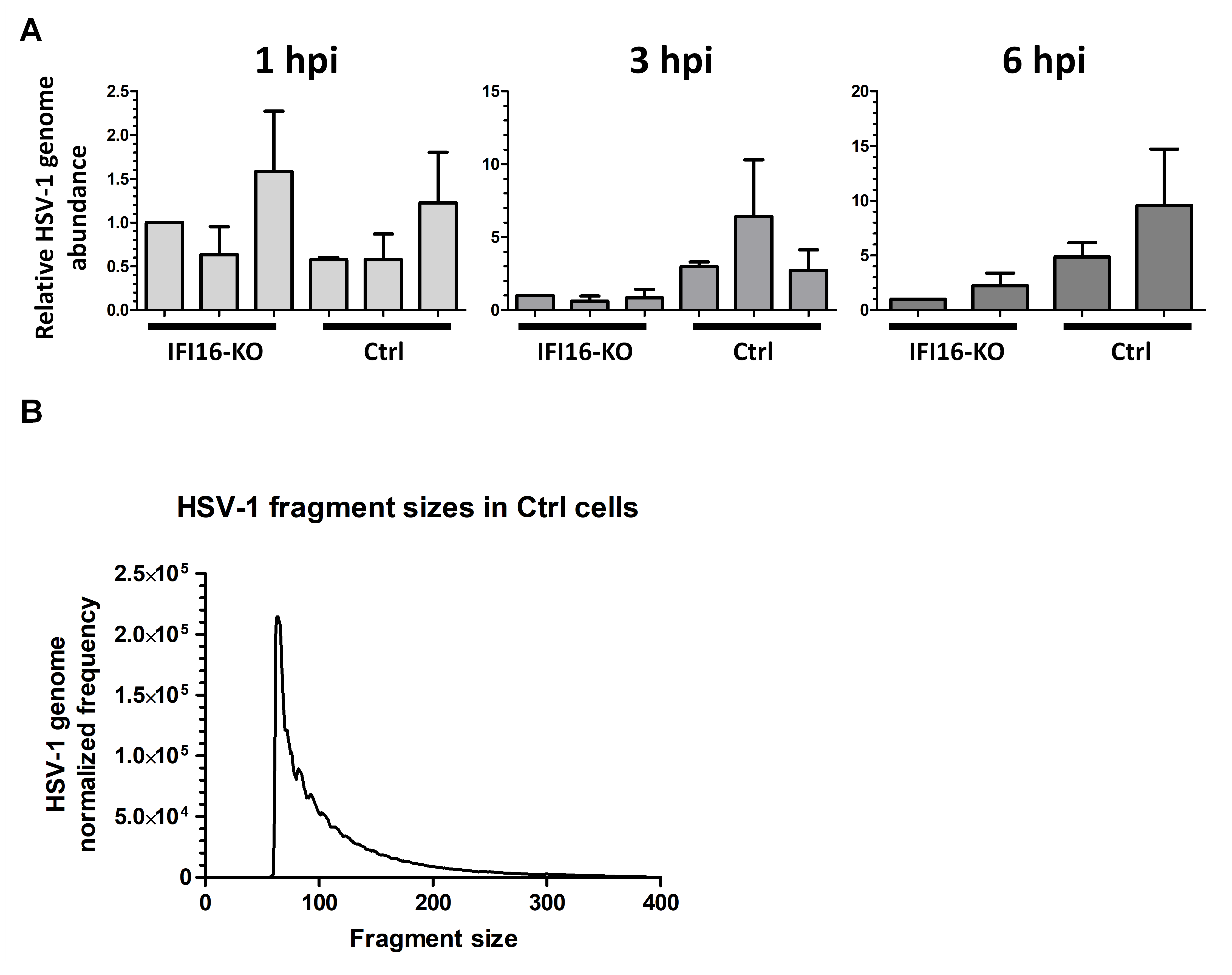

Supplement: FIG S2 [file msystems.00198-22-s0003.tif]

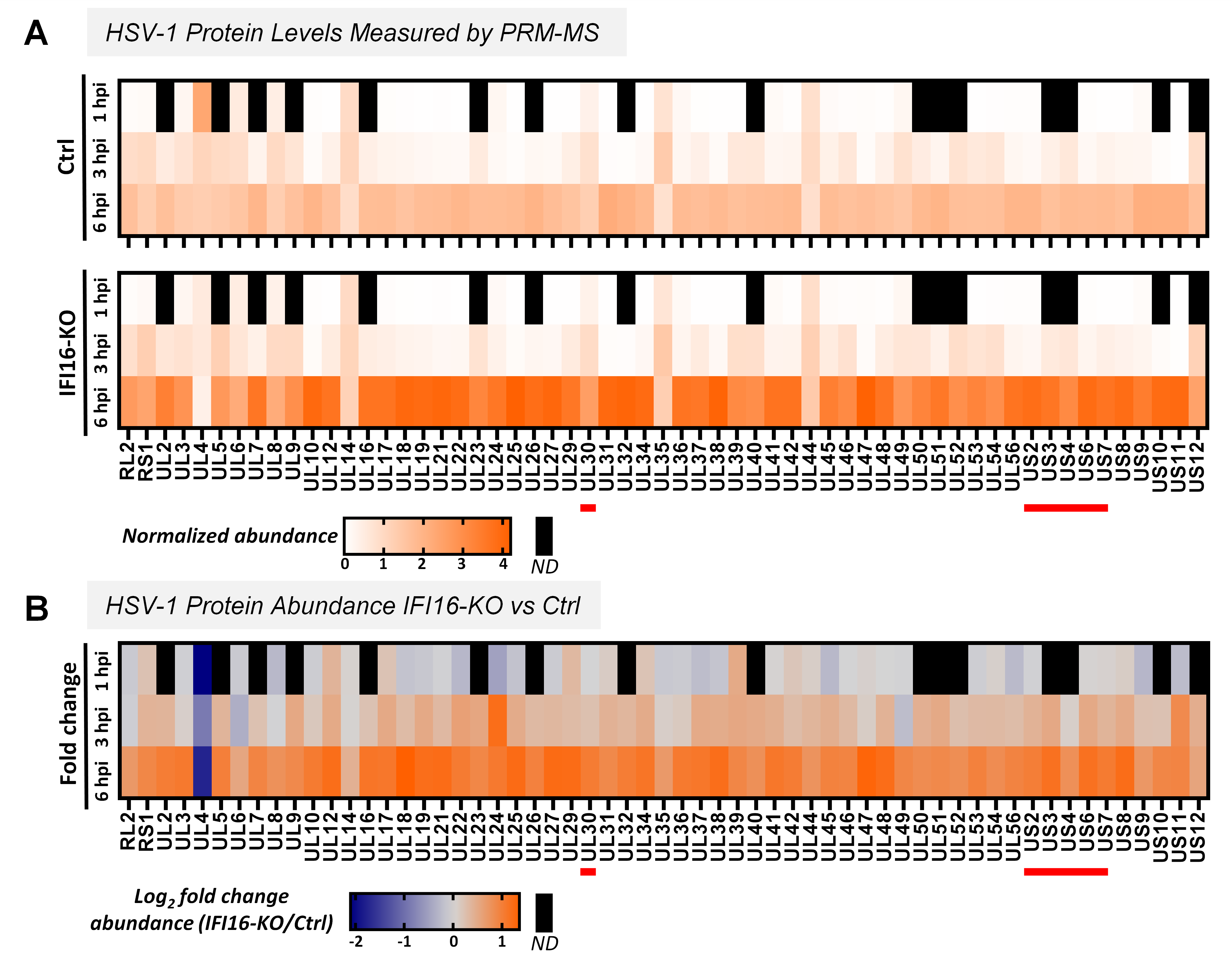

Supplement: FIG S3 [file msystems.00198-22-s0004.tif]

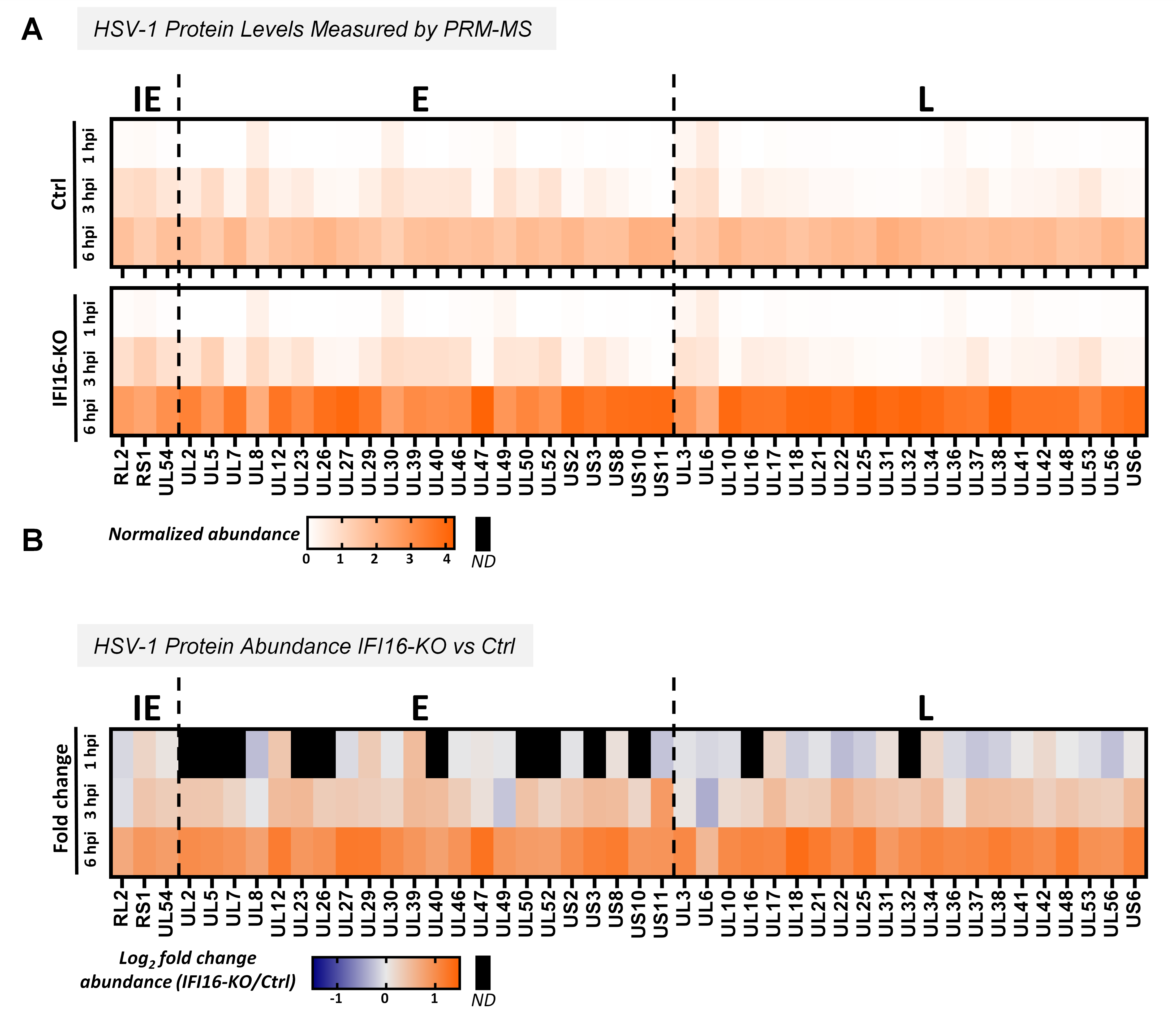

Supplement: FIG S4 [file msystems.00198-22-s0005.tif]

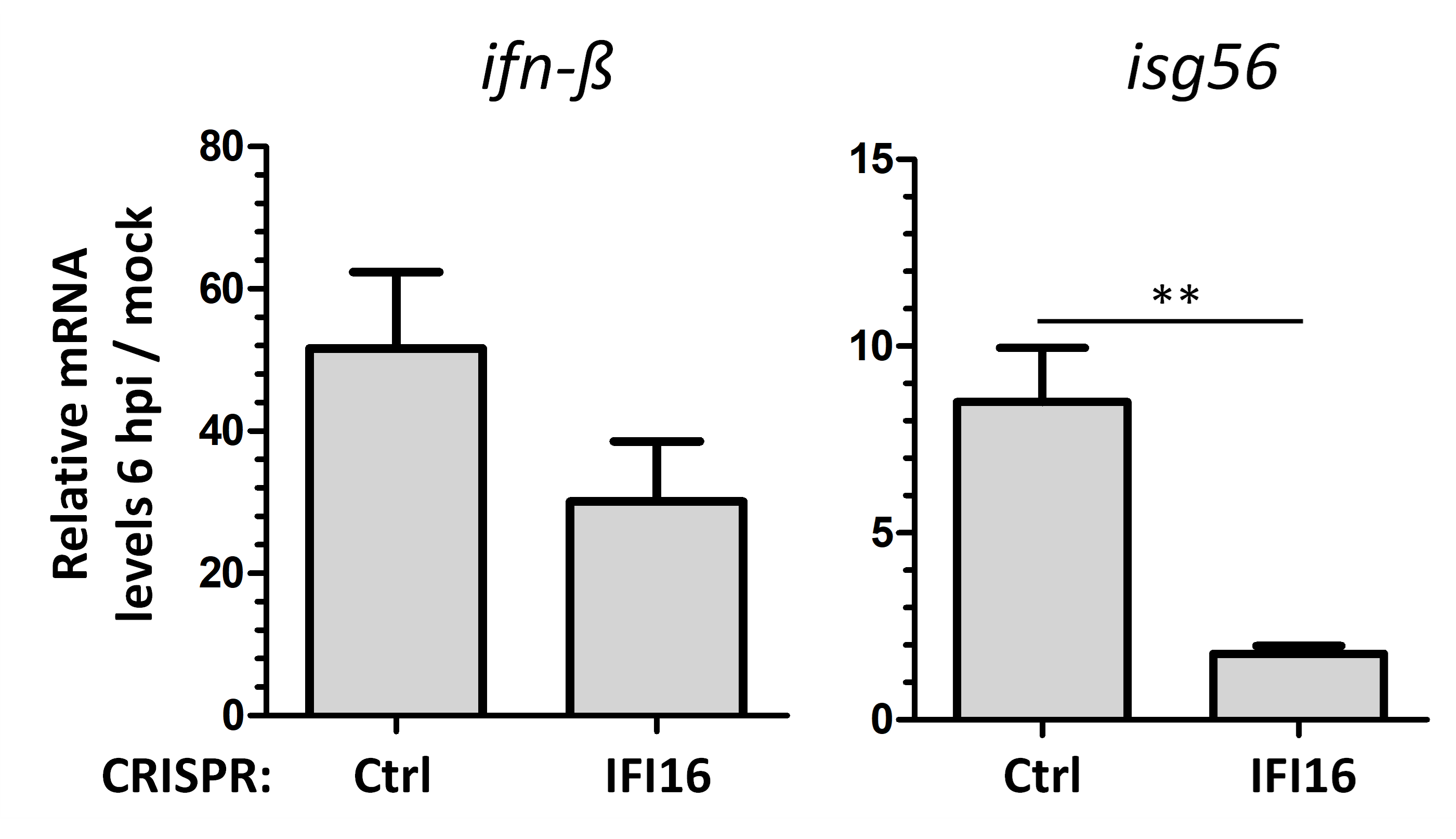

Supplement: FIG S5 [file msystems.00198-22-s0006.tif]
